# OA04 Multifocal sclerotic bone lesions mimicking metastatic disease: a diagnostic pitfall in atypical Paget’s disease of bone

**DOI:** 10.1093/rap/rkag113.004

**Published:** 2026-11-03

**Authors:** Rehab Mohammed, Aabha Sinha, Holly John

**Affiliations:** Rheumatology, The Dudley Group, Dudley, United Kingdom; Radiology, The Dudley Group, Dudley, United Kingdom; Rheumatology, The Dudley Group, Dudley, United Kingdom

## Abstract

**Introduction:**

Paget’s disease of bone is a chronic disorder of disordered bone remodelling that may be asymptomatic or present with non-specific musculoskeletal symptoms. In atypical or multifocal presentations, radiological findings may closely resemble osteoblastic metastatic disease, resulting in significant diagnostic uncertainty. Differentiating metabolic bone disease from disseminated malignancy is essential to avoid unnecessary invasive investigations and inappropriate oncological treatment. We report a case of incidentally detected multifocal sclerotic bone lesions initially suspected to represent metastatic malignancy, ultimately diagnosed as Paget’s disease following comprehensive multidisciplinary evaluation.

**Case description:**

A 51-year-old woman presented with abdominal pain due to an irreducible epigastric hernia. CT abdomen performed for surgical assessment incidentally demonstrated multiple sclerotic lesions throughout the axial skeleton, raising concern for metastatic malignancy.

She had no constitutional symptoms and no known malignancy. A family history of Paget’s disease of bone was present. Biochemical testing revealed elevated alkaline phosphatase (225 IU/L).

Extensive investigations to exclude occult malignancy were undertaken, including mammography, CT chest, thyroid ultrasound, colonoscopy, PET-CT, and MRI spine, all of which were negative.

Bone scintigraphy demonstrated multifocal increased uptake, with a differential diagnosis of metastatic disease versus metabolic bone disorder. Radiological reassessment identified evolving features more suggestive of Paget’s disease, including involvement of the skull and clavicle.

Given persistent diagnostic uncertainty, bone marrow biopsy was performed and showed no evidence of malignancy.

A final diagnosis of Paget’s disease of bone was made based on characteristic imaging evolution, biochemical findings, and multidisciplinary consensus. The patient was treated with a single infusion of zoledronic acid, resulting in marked biochemical improvement consistent with disease control.

**Discussion:**

This case highlights the diagnostic challenge posed by incidentally detected multifocal sclerotic bone lesions and reinforces the importance of considering Paget’s disease in the differential diagnosis of suspected metastatic malignancy.

Although classical Paget’s disease has recognisable radiological features, atypical multifocal presentations may closely mimic osteoblastic metastases, particularly when detected incidentally. Imaging alone may be insufficient for diagnosis, requiring integration with biochemical, clinical, and evolving radiological findings.

In this case, the absence of systemic red flag symptoms prompted initial broad malignancy work-up. While multimodal imaging refined the differential diagnosis, it did not definitively exclude metastatic disease, highlighting the limitations of imaging alone in complex skeletal presentations.

Bone marrow biopsy was useful in excluding malignancy when uncertainty persisted. Final diagnosis was achieved through multidisciplinary correlation of imaging evolution, biochemical response, and clinical assessment.

The marked biochemical response to zoledronic acid further supported the metabolic nature of the disease and confirmed disease control.

This case underscores the importance of a structured multidisciplinary approach to incidental multifocal skeletal lesions and highlights how metabolic bone disease can closely mimic disseminated malignancy.

**Key learning points:**

1. Incidentally detected multifocal sclerotic lesions may closely mimic metastatic malignancy, requiring systematic diagnostic evaluation.

2. Bone scintigraphy and PET-CT may not reliably distinguish metabolic bone disease from skeletal metastases.

3. Imaging interpretation may evolve with multidisciplinary reassessment, particularly in atypical or incidentally detected disease.

4. Bone marrow biopsy can be valuable in excluding malignancy when imaging findings remain inconclusive.

5. Diagnosis of Paget’s disease may depend on integration of imaging evolution, biochemical response, and multidisciplinary consensus.

6. Biochemical improvement following bisphosphonate therapy may support diagnostic confidence and therapeutic response.

